# YOU’VE BEEN CATFISHED: An exploration of social deception on online platforms

**DOI:** 10.1192/j.eurpsy.2022.1477

**Published:** 2022-09-01

**Authors:** S. Jesus, A. Costa, G. Simões, G. Dias Dos Santos, M. Almeida, P. Garrido

**Affiliations:** 1 Baixo Vouga Hospital Centre - EPE, Psychiatry And Mental Health Department, Aveiro, Portugal; 2 Centro Hospitalar do Baixo Vouga, Psiquiatria E Saúde Mental, Aveiro, Portugal

**Keywords:** social interaction, personality, e-mental health, catfish

## Abstract

**Introduction:**

Life is a stage in which we are all actors and online we can choose who we want to be. Catfishing is a modern phenomenon in which individuals present themselves online as someone they are not as a means of engaging with others through an idealized avatar. This term has gained prominence since its portrayal in documentary and television series. With the emergence of catfishing, an expectation of betrayal in online relationships is anticipated with increasing caution being exercised by those that engage in online forums.

**Objectives:**

The authors aim to explore this phenomenon and explore what personality traits might be associated with those who engage in catfishing others and in those that fall for the dupe.

**Methods:**

A review of the recent literature on the topic with focus on that which is most relevant to the theme was included.

**Results:**

The literature demonstrates that catfishing is an increasing trend as our online social interaction also increases. Catfishing appears to exist on a scale, where approximately 80% of the online population engage in some form, by means of amplifying their social status. Those with low self-esteem, poor self-worth with and a need to connect and to be validated were most susceptible.

**Conclusions:**

The internet permits anonymity where trading “real world” skins with digital ones creating avatars in order to seek what is desired. Catfishing raises questions about the nature of the human self and the role it plays in deception. Understanding how patients use the internet may provide insight into how personality acts on a stage of total anonymity.

**Disclosure:**

No significant relationships.

